# Facilitators and Reducers of Korean Travelers’ Avoidance/Hesitation Behaviors toward China in the Case of COVID-19

**DOI:** 10.3390/ijerph182312345

**Published:** 2021-11-24

**Authors:** Heesup Han, Chen Che, Sanghyeop Lee

**Affiliations:** 1 College of Hospitality and Tourism Management, Sejong University, Seoul 143-747, Korea; heesup.han@gmail.com; 2 College of History and Tourism Culture, Inner Mongolia University, Hohhot 010021, China; cecencan@naver.com; 3 Major in Tourism Management, College of Business Administration, Keimyung University, Daegu 42601, Korea

**Keywords:** risk perception, China as an international tourism destination, anticipated emotions, destination attachment, post-pandemic

## Abstract

Given that little is known about overseas travelers’ responses and behaviors toward China after the outbreak of COVID-19, this study aimed to uncover risk perception factors and investigate its role in Korean travelers’ avoidance/hesitation behaviors toward China as an international tourism destination in the case of the COVID-19 pandemic. To explore the relationship with risk perception, anticipated emotion and avoidance/hesitation behavior, a quantitative method along with an online survey was employed. This focus was on Korean tourists who had traveled to China at least once. Findings revealed that risk perception and negative anticipated emotion are vital facilitators of avoidance/hesitation behaviors, and that positive anticipated emotion reduces such behaviors. The efficacy of a higher-order structure of risk perception, which encompasses six dimensions, was also demonstrated. In addition, destination attachment lowered the influence of risk perception on the formation of avoidance/hesitation behaviors. Overall, our results will help tourism researchers and practitioners understand what factors drive and reduce international travelers’ avoidance/hesitation behaviors toward China in the post-pandemic world. Implications for theory and practice are offered.

## 1. Introduction

The impact of a coronavirus diseases 19 (COVID-19) on the entire world is substantial [1,2,3]. The pathogenic influence of COVID-19 has been drastically increased since China reported the first confirmed case in late 2019 [4,5]. This virus fast became a huge threat to human health [6]. Moreover, ever since the detection of the COVID-19, this disease has considerably affected human mobility [7]. COVID-19 had a huge adverse effect on the tourism sector [1,8]. Particularly, the international tourism industry has been massively reduced due to border closing, lockdowns, and bans on traveling [3,5]. The COVID-19 characteristics of high prevalence, broad distribution, and geographical variables made this situation worse for the tourism industry [3,4,7].

Until 11 November 2021, the World Health Organization (WHO) reported that there had been 25.127 million confirmed cases of COVID-19 in the world, including 0.507 million deaths [9]. In China, there have been 3283 confirmed cases of COVID-19 [10]. Most overseas travelers have inevitably restrained themselves from visiting international tourism destinations until the pandemic is entirely under control [1,8]. COVID-19 has also blocked international tourists’ visit to China. The global concern for human health derived from the pandemic significantly reduced international tourism demand throughout the world. According to Chen et al. [11], there is a high possibility that another new type of coronavirus will emerge in the future because of rapid climate change, ecological problems, fast-increasing flows of human populations, and increase in human–animal interactions. Indeed, many countries are shifting to the with-corona era.

Irrefutably, there exists a critical association between COVID-19 and tourist responses/behavior [7,8]. Yet scant research has uncovered the possible influence of perceived risk pertinent to the disease on overseas travelers’ anticipated emotions and avoidance responses to traveling to China. Especially, very little is known about Korean international travelers’ responses and behaviors toward traveling to China since the emergence of COVID-19. For Korean travelers, China is one of the preferred destination countries. Indeed, the statistics of the Korea Tourism Organization [12] showed that about 4,346,567 Koreans visited China in 2019 before the outbreak of the pandemic. They traveled to China for diverse purposes (wellness, sightseeing, reputation/image of local destinations, low price, shopping, leisure, and foods) [12]. Undoubtedly, the image and popularity of China as a tourism destination has been considerably influenced by COVID-19. Exploring Korean international tourists’ perception of traveling to China in the post-pandemic world is vital to better understand their emotional tendencies and behaviors.

The objectives of this study were therefore to identify Korean travelers’ perception regarding possible risks of traveling to China in the post-pandemic world and to explore the influence of such risk perception and its dimensions (i.e., human crowding risk, spatial crowding risk, quality risk, psychological risk, health and safety risk, and financial risk) on the formation of avoidance/hesitation behaviors. In addition, this research aimed to uncover the efficacy of a higher-order structure of risk perception. Moreover, this study was designed to unearth the moderating impact of destination attachment and to investigate the mediating effect of positive and negative anticipated emotions. Lastly, this research assessed the impact of Korean travelers’ avoidance/hesitation behaviors in the COVID-19 era.

## 2. Literature Review

### 2.1. Risk Perception and Its Role

The criticality of risk perception has long been emphasized in various sectors due to its considerable influence on individuals’ behaviors [13,14,15]. Particularly in the hospitality/tourism literature, risk perception and its importance are extensively stressed [13,16,17]. Undoubtedly, risk perception that has multiple dimensional characteristics is the major constituent of traveler approach/avoidance decision formation [17,18]. The vital facet of risk perception in tourism as well as in consumer behavior and psychology is uncertainty and an individual’s apprehension of it [15,19]. Such uncertainty is negatively linked to anticipated responses from individuals [19]. The critical aspects of risk perception in an unsafe consumption environment (e.g., the pandemic world) can be crowding risk (human and spatial), quality risk, psychological risk, health and safety risk, and financial risk [15,16,18,20]. Given this, the concept of risk perception in the present research indicates travelers’ uncertainty about tourism-destination performances and their cognitive anxiety regarding the disparity between such performances and travelers’ expectations.

An extensive amount of the extant literature has assessed the possible effect of risk perception on individuals’ approach/avoidance responses and behaviors [14,17,21]. Olya and Altinay [22] explored how risk perception influences traveler post-purchase behaviors either in a positive or negative way. In their investigation on international travelers’ behaviors, Al-Ansi et al. [13] found risk perception as a vital contributor to behavioral intention generation. More interesting in this research is how to manage consumers’ perceived risk to improve their satisfaction and trust. Law [23] examined travelers’ decision formation for overseas destination choices when there is any probability of the occurrences of virus infection, terrorist attack, and natural disasters. He found that ones’ international tourism decision is significantly affected by risk perception level. Scholars in recent research have also revealed that risks related to crowdedness, quality/performance, mental health/personal psychology, physical health/safety, and money are of importance as such risks elicit positive and negative emotional evaluations and avoidance/hesitation behaviors [16,17,20]. Olya and Al-Anish [16] examined how health risk, quality risk, and psychological risk positively influence tourist satisfaction, and financial risk significantly affected tourist behavioral intention. Yu et al. [17] point out the risk perception (psychological and financial risk) of COVID-19, which strongly negatively influences tourist behavioral intention. The studies discussed above support the possible linkages among risk perception, anticipated emotional responses, and avoidance/hesitation behaviors. In addition, the finding of a recent study evidenced the efficacy of a second-order structure of risk perception in the tourism context [13]. Therefore, we developed hypotheses as follows:

**Hypothesis** **1** **(H1).** *Risk perception has a significant influence on positive anticipated emotion*.

**Hypothesis** **2** **(H2).** *Risk perception has a significant influence on negative anticipated emotion.*

**Hypothesis** **3** **(H3).** *Risk perception has a significant influence on avoidance/hesitation behavior.*

### 2.2. Anticipated Emotions

For the last few decades, anticipated emotions have been an important concept in customer behavior and tourism [24,25]. Anticipated emotions are described as anticipated affective process. Perugini and Bagozzi [26] indicate the prospect of feeling favorable or unfavorable affects when conducting or not conducting a particular action [27]. This conceptualization is in line with Han [28] who described these anticipated emotions are one’s expected positive/negative feelings after performing or not performing a specific action. In general, researchers agree that anticipated emotions have two dimensions (positive and negative) [28,29,30]. These positive and negative forms of anticipated affective responses are believed to be important determinants of consumer behaviors [25].

There has been a significant improvement in extant socio-psychological theories/models of anticipated emotion, such as the theory of planned behavior, norm activation theory, and the value-belief-norm model [24,26,30]. Rosenthal and Ho [24] found that personal norms have a mediating effect on anticipated negative emotions and awareness of consequences. According to their two studies, Perugini and Bagozzi [26] demonstrated that anticipated emotions are an essential factor of desire. Lu et al. [30] distinguish anticipated guilt and anticipated pride in analyzing employees’ pro-environment behavior. Indeed, theoretical/conceptual frameworks encompassing positive and negative forms of anticipated emotion, which were designed for self-interest/pro-social consumer behaviors, have benefited from the increase in prediction power for consumer approach/avoidance responses and behaviors [26,28]. The inclusion of positive and negative anticipated emotions often increases the ability of a theoretical model in explicating individuals’ behaviors in a product-purchase or consumption situation [29,31]. Findings of the existing studies in the extant literature have also revealed that positive and negative anticipated emotions play a crucial role in inducing avoidance or approach behaviors for products/services [24,31]. Therefore, we developed hypotheses as follows:

**Hypothesis** **4** **(H4).** *Positive anticipated emotion has a significant influence on avoidance/hesitation behavior*.

**Hypothesis** **5** **(H5).** *Negative anticipated emotion has a significant influence on avoidance/hesitation behavior*.

### 2.3. Destination Attachment

Destination attachment has received increasing attention from tourism marketers and academics since it contributes considerably to enhancing travelers’ favorable decisions/behaviors regarding a tourism destination (retention, loyalty behaviors, protection behaviors, word-of-mouth, sustainable actions at a destination) [24,32,33,34]. Destination attachment is a concept that describes the emotional tie between visitors and place [35]. Similarly, Rosenthal and Ho [24] described destination attachment as an affective bond between the place and its visitors. This concept is often an alternative for terms such as sense of belonging and involvement [28].

Sohn and Yoon [36] identified five characteristics of risk perception (social, physical, financial, health and psychological risk). They argued that destination attachment had a moderate influence on risk perception (physical and health) and destination image. Pangaribuan et al. [37] demonstrated that risk perception had a moderate effect on destination attachment and voluntary behavior. In general, risk perception is important for tourist avoidance/hesitation behavior [38]. According to Pradhananga and Davenport [34] and Fournier and Lee [32], a traveler with a strong attachment to a certain destination will more actively practice loyalty and citizenship behaviors regarding that destination (e.g., volunteering, repeat visit, recommendation). Indeed, when visitors’ level of destination attachment is high, they are likely to show positive emotional responses, feel involvement, and show approach behaviors towards the destination [28,32,34,35]. Undoubtedly, destination attachment is a significant contributor influencing the formation of emotional reactions and behavioral responses [24]. The contributing role of attachment/involvement as a moderator is evident in consumer behavior and tourism [32,33]. Accordingly, the following hypotheses were developed:

**Hypothesis** **6a** **(H6a).** *Destination attachment has a moderating influence on relation between risk perception and positive anticipated emotion*.

**Hypothesis** **6b** **(H6b).** *Destination attachment has a moderating influence on relation between risk perception and negative anticipated emotion*.

**Hypothesis** **6c** **(H6c).** *Destination attachment has a moderating influence on relation between risk perception and avoidance/hesitation behavior*.

### 2.4. Theoretical Model

**Figure 1:**
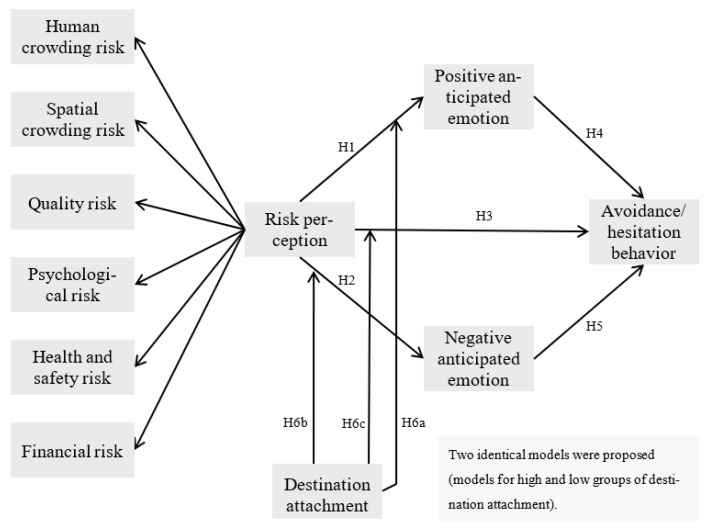
The proposed model.

The research model is exhibited in Figure 1. Our proposed framework contains risk perception and anticipated emotions (positive and negative) as predictors of traveler avoidance/hesitation behaviors and encompasses destination attachment as a moderator. Human crowding risk, spatial crowding risk, quality risk, psychology risk, health and safety risk, and financial risk are incorporated as the first-order factors of risk perception. In addition, the model includes six research hypotheses within its theoretical framework.

## 3. Methods

### 3.1. Measurement Development

The measures of risk perception and its dimensions are generated based on a thorough review of the literature [13,15,39,40,41]. Additionally, several measurement items were added on the basis of qualitative interviews (i.e., “The flow of people in the tourist areas of China is slow because of too many people”, “Hotels/restaurants/shopping places are in general not spacious enough”, “I am worried that the quality of tourism products in China will be lower after the COVID-19 pandemic ends in the near future”, and “The thought of traveling to China causes me to experience unnecessary tension although the COVID-19 pandemic may end in the near future”). The person-to-person interviews were conducted with actual Korean travelers who often visited China for international tourism before the outbreak of the pandemic. Overall, three items were utilized to measure human crowding risk (i.e., “Too many people are in tourist sites in China”, “Because of the huge number of tourists, visitors feel crowded in many tourist sites in China”, “The flow of people in the tourist areas of China is slow because of too many people”), and three items were employed to assess spatial crowding risk (i.e., Because of spatial crowdedness, visitors often feel stuffy in many tourist sites in China”, “Due to little space, moving around is not easy in many of the tourist sites of China”, “Hotels/restaurants/shopping places are in general not spacious enough”. Additionally, two items and three items were utilized to measure quality risk (i.e., “I am concerned with the lower quality of tourism products in China after the COVID-19 pandemic ends in the near future”, “I am worried that the quality of tourism products in China will become lower after the COVID-19 pandemic ends in the near future”. and psychological risk (i.e., “The thought of traveling to China makes me feel anxious although the COVID-19 pandemic may end in the near future.”, “The thought of traveling to China makes me feel psychologically uncomfortable although the COVID-19 pandemic may end in the near future”, “The thought of traveling to China causes me to experience unnecessary tension although the COVID-19 pandemic may end in the near future”), respectively. Health and safety risk (i.e., “Traveling to China, although the COVID-19 pandemic may end in the near future, is still unsafe”, “Traveling to China, although the COVID-19 pandemic may end in the near future, is still unhealthy”, “Traveling to China, although the COVID-19 pandemic may end in the near future, is still risky”) and financial risk (i.e., “I am afraid there will be considerable extra expenses in many tourist sites in China after the COVID-19 pandemic ends in the near future”, “I worry that visiting China will involve unexpected extra expenses after the COVID-19 pandemic ends in the near future”) were measured with three items and two items, respectively.

The measures for other constructs were taken from the existing studies [26,31,32,42,43]. Specifically, destination attachment was assessed with three items. Four items were used to measure positive anticipated emotions whereas three items were used to evaluate negative anticipated emotions. Moreover, avoidance/hesitation behavior was measured with five items. That is, multiple measures with a seven-point Likert-scale for all constructs were used in the present research.

### 3.2. Data Collection Procedure and Sample Characteristics

To test the proposed hypothesized framework, this study employed a Web-based survey method. Using an online market research firm’s system, the survey link was generated. Afterwards, using social media (e.g., kakaotalk, Instagram), this was frequently used by Korean people to send this link to potential survey participants who had traveled to China at least once. Before sending the questionnaire link, respondents were asked about their willingness to participate and their experience of visiting China. The qualified survey participants were asked to read thoroughly the study description and questions when filling out the questionnaire. To complete this survey, the participants had to answer all inquiries in the questionnaire. A total of 450 survey questionnaires was collected. Among them 429 usable responses were gathered through this process. These cases were used for data analysis.

Among 429 survey participants, 50.6% were male travelers whereas 49.4% were female travelers. The participants’ age fell between 20–73 years old with a mean age of 40.1 years. Specifically, about 23.3% were less than 29 years old; 25.6% were between 30–39 years old; 24.7% were between 40–49 years old; and 26.3% were above 50 years old. About 94.6% of the participants reported that they had visited China within the last three years. All respondents had visited to China within the last 5 years. Regarding education level, 69.2% were college graduates, followed by graduate-degree holders (16.8%), high-school graduates (9.3%), and others (4.7%). About 56.9% of the survey participants reported that they were married whereas 43.1% reported other forms of marital status. In terms of annual income level, about 38.9% indicated an income between 30,000–50,000 USD, followed by less than 30,000 USD (28.0%), 50,000–80,000 USD (23.3%), and over 80,000 USD (9.8%).

## 4. Results

### 4.1. Reliability and Validity Assessment

A confirmatory factor analysis was performed to create a measurement model. The model assessment revealed that a satisfactory model fit (χ^2^ = 969.977, *df* = 387, *p* < 0.001, χ^2^/*df* = 2.506, RMSEA = 0.059, CFI = 0.963, IFI = 0.963, TLI = 0.956). All items were loaded to their relevant latent factor in a significant manner (*p* < 0.01). As shown in Table 1, all composite reliability values (human crowding = 0.925; spatial crowding = 0.831; quality risk = 0.955; psychological risk = 0.946; health and safety risk = 0.959; financial risk = 0.914; positive anticipated emotion = 0.957; negative anticipated emotion = 0.885; destination attachment = 0.969; and avoidance/hesitation behavior = 0.967) exceeded the suggested threshold of 0.70 [44]. This result evidenced internal consistency of the multiple-item measures. Average variance extracted values were calculated. All values (human crowding = 0.804; spatial crowding = 0.622; quality risk = 0.913; psychological risk = 0.853; health and safety risk = 0.886; financial risk = 0.841; positive anticipated emotion = 0.846; negative anticipated emotion = 0.720; destination attachment = 0.913; and avoidance/hesitation behavior = 0.853) exceeded the recommended threshold of 0.50 [44]. In addition, the values were greater than between-construct correlations (squared), as evidenced in Table 1. This evidenced the convergent and discriminant validity of the multiple-construct measures.

### 4.2. Structural Model Assessment and Hypothesis Testing

**Figure 2:**
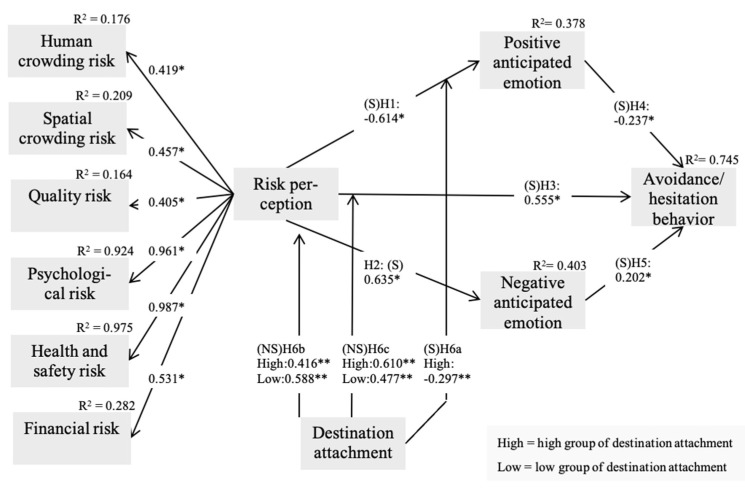
Structural model and invariance model results. Note1. Goodness-of-fit statistics for the structural model (higher-order model χ^2^ = 1079.063, *df* = 337, *p* < 0.001, χ^2^/*df* = 3.202, RMSEA = 0.072, CFI = 0.946, IFI = 0.946, TLI = 0.939. Note2. Goodness-of-fit statistics for the baseline model: χ^2^ = 1567.221, *df* = 693, *p* < 0.001, χ^2^/*df* = 2.262, RMSEA = 0.054, CFI = 0.925, IFI = 0.925, TLI = 0.918. Note3. * *p* < 0.05, ** *p* < 0.01. Note4. Two identical models were proposed (models for high and low groups of destination attachment).

A structural equation modeling was conducted with the use of Maximum likelihood estimation (see Table 2). The result demonstrated a satisfactory model fit (χ^2^ = 1079.063, *df* = 337, *p* < 0.001, χ^2^/*df* = 3.202, RMSEA = 0.072, CFI = 0.946, IFI = 0.946, TLI = 0.939) (see Figure 2). The second-order model for risk perception indicates that the global higher-order factor is significantly related to the six first-order variables (human crowding, spatial crowding, quality risk, psychological risk, health and safety risk, and financial risk). The coefficients were 0.419 (*p* < 0.01) for human crowding risk, 0.457 (*p* < 0.01) for spatial crowding risk, 0.405 (*p* < 0.01) for quality risk, 0.961 (*p* < 0.01) for psychological risk, 0.987 (*p* < 0.01) for health and safety risk, and 0.531 (*p* < 0.01) for financial risk, respectively. The higher-order latent variable accounted for about 17.6%, 20.9%, 16.4%, 92.4%, 97.5%, and 28.2% of the total variance for human crowding, spatial crowding, quality risk, psychological risk, health and safety risk, and financial risk, respectively. This evidenced the adequacy and effectiveness of the higher-order structure of risk perception within the proposed theoretical framework.

The hypothesized influence of risk perception on anticipated emotions and avoidance/hesitation behavior was tested. Our findings showed that risk perception exerted a significant influence on positive anticipated emotion (β = −0.614, *p* < 0.01) and negative anticipated emotion (β = 0.635, *p* < 0.01). Additionally, risk perception affected avoidance/hesitation behavior significantly (β = 0.555, *p* < 0.01). Hypotheses 1, 2, and 3 were hence supported. The proposed effect of anticipated emotions was assessed. Results revealed that both positive anticipated emotion (β = −0.237, *p* < 0.01) and negative anticipated emotion (β = 0.202, *p* < 0.01) had a significant impact on avoidance/hesitation behavior. Therefore, Hypotheses 4 and 5 were supported. About 37.8% and 40.3% of the variance in positive and negative anticipated emotions were accounted for by risk perception. In addition, these variables explained 74.5% of the total variance in avoidance/hesitation behavior. A close examination of indirect relationship showed that risk perception contained a significant indirect influence on avoidance/hesitation behavior through positive and negative anticipated emotions (β = 0.274, *p* < 0.01). The total impact of risk perception on avoidance/hesitation behavior (β = 0.828, *p* < 0.01) was the greatest among study variables. Overall, these results indicate that risk perception (e.g., human crowding risk, spatial crowding risk, quality risk, psychological risk, health and safety risk, financial risk) has a positive relationship with anticipated emotions (positive and negative anticipated emotion) and avoidance/hesitation behavior.

### 4.3. Baseline Model and Test for Metric Invariance

A baseline model was created to evaluate the hypothesized moderating influence of destination attachment. A total of 429 responses gathered through the survey were divided into high destination attachment group (*n* = 198) and low destination attachment group (*n* = 231). We utilized a k-means cluster analysis for the grouping process. Findings showed that this baseline model contained a satisfactory model fit (χ^2^ = 1567.221, *df* = 693, *p* < 0.001, χ^2^/*df* = 2.262, RMSEA = 0.054, CFI = 0.925, IFI = 0.925, TLI = 0.918). The baseline model results are shown in Table 3.

The generated baseline model was compared to the nested models, where a particular link of interest was constrained equivalently across high and low destination attachment groups. The results of a Chi-square test indicated that the linkage from risk perception to positive anticipated emotion significantly differed across groups (Δχ^2^ (1) = 3.888, *p* < 0.05). Therefore, Hypothesis 6a was supported. Yet findings revealed that the paths from risk perception to negative anticipated emotion (Δχ^2^ (1) = 2.712, *p* > 0.05) and to avoidance/hesitation behavior (Δχ^2^ (1) = 0.772, *p* > 0.05) were not significantly dissimilar between destination attachment groups. Accordingly, Hypotheses 6b and 6c were not supported. In summary, these results indicate that destination attachment has positive moderating effect on risk perception and avoidance/hesitation behavior. Conversely, the destination attachment was not a moderating effect on risk perception and anticipated emotions (positive and negative anticipated emotion).

## 5. Discussion

This study provides a meaningful theorization related to tourist international behaviors in the with-corona era. The present research is one of few studies that have built a theoretical framework by risk perception factors as core variables and considered their influence on international travelers’ avoidance behavior through anticipated emotions. This research aimed to integrate the moderating impact of overseas travelers’ destination attachment with the formation of their anticipated emotional responses and hesitation behaviors. In sum, the findings of the present research help us to comprehend more explicitly the role of risk perception, which increases international travelers’ negative anticipated emotion, decreases their positive anticipated emotion, and ultimately enhances their avoidance actions regarding travel to China. In addition, our findings help us to clearly understand the criticality of attachment and how it moderates the risk perception and positive anticipated emotion relationship. Given that international tourism toward China can be one of the most critical issues in the global tourism industry in the with-corona era, the findings of the present research are of utmost importance in helping international tourism practitioners and researchers in Korea enhance their knowledge about outbound travelers’ decision-making processes and behaviors in relation to China in the with-corona era.

It is indisputable that there exists a significant relation between disease outbreak and tourist behaviors. Nevertheless, little research has yet uncovered the possible effect of perceived risk related to COVID-19 on Korean travelers’ destination choices in the outbound tourism sector. Filling this void, this is the first empirical study that identifies the possible risks of traveling to China after the outbreak of COVID-19 and explores the influence of various risk factors on Korean travelers’ avoidance behaviors/responses toward traveling to China. Our result therefore enriches the literature on international tourism and can be used as a basic framework for future research about disease outbreak and tourist behavior.

Al-Ansi [13] determined the risk perception dimension of health risk, psychological risk, environmental risk, social risk, quality risk, financial risk, and time-loss risk and found that, except for time-loss risk, risk perception significantly influenced tourist behavioral intention. In the current research, a particular meaningful point is the second-order framework of risk perception. It was apparent that the six first-order dimensions (human crowding risk, spatial crowding risk, quality risk, psychological risk, health and safety risk, and financial risk) belong to the global latent factor of risk perception. In other words, the commonality underlying the six dimensions was fully extracted by its second-order variable. This empirical result enriches the extant literature by providing a higher-order approach, which clearly captures risk perception pertinent to international tourism in China. Moreover, the parsimonious framework of this hierarchical structure enlightens academics and destination marketers regarding the effectiveness of theorizing intricate risk perception factors more succinctly in the with-corona era of the international tourism sector. For sector can ensure social distancing in tourism destinations to avoid human crowding and spatial crowding risk. Zhang et al. [45] suggested that destination managers should weigh up destination measures and tourist health risk perception, and that destination anti-epidemic measures positively influence destination image. Combined with this research, tourism departments need to improve this destination management measure.

In a previous study on the relationship between health risk and tourist protective behavior, worry played a crucial role in health risk perceptions [17,46]. A prior study confirmed the relationship between financial and quality risk and tourist behavioral intention [11,16]. In this research health and safety risk were two prominent factors of risk perception. Therefore, for tourism practitioners in China, improving unhealthy, unsafe, and uncomfortable tourism environments can be crucial to minimize risk perception of Korean international travelers regarding China.

Financial risk and quality risk were also uncovered as vital dimensions of risk perception. In addition, human crowding risk and spatial crowing risk were unearthed as significant dimensions of risk perception. Hence, lowering financial and quality risks in tourist sites in China, as well as minimizing human and spatial crowding risks, are critical. The facilitators and inhibitors of tourist avoidance behaviors relating to international tourism are weakly researched and understood in the with-corona era. Employing an empirical approach, this research successfully provides evidence that risk perception and its constituents are fundamental sources for helping tourism academics/practitioners in Korea and China in understanding outbound and inbound travelers’ responses/behaviors, respectively.

According to prior research, tourists’ destination attachment strongly influences destination loyalty and positive emotional response [32,34,35]. The findings of our research from metric-invariance assessment evidenced that destination attachment significantly moderates the relation between risk perception and positive anticipated emotion. Particularly, the risk perception→ positive emotion relationship was weaker in the high group of destination attachment (β_risk perception__→__positive anticipated emotion_ = −0.297, *p* < 0.01) as compared to the low group (β_risk perception__→__positive anticipated emotion_ = −0.481, *p* > 0.05). Our result indicates that Korean overseas travelers’ risk perception is less likely to reduce their positive anticipated emotion when they feel a strong attachment to China as an international tourism destination. Sohn and Yoon [36] reported that destination attachment has a moderate effect on risk perception and destination image. The result of this research evidence provides theoretically important information that the risk perception and positive anticipated emotion link significantly depends on the level of destination attachment. The use of this moderator concept can hence be hence for a better grasp of understanding Korean tourists’ avoidance responses when considering China for international tourism. Practically, our result offers critical insights as it reports that strong bonding between a destination and its visitors reduces the detrimental influence of risk perception on otherwise positive anticipated emotional responses. 

## 6. Conclusions

Our investigation of the indirect influence of research constructs evidenced that positive and negative anticipated emotions are crucial mediators within the hypothesized conceptual framework. Two main influential factors mediated the impact of their proximal antecedent to their outcome construct. The result indicates that for a clear understanding of the role of risk perception in overseas travelers’ behaviors, dealing with positive and negative anticipated emotions is a fundamental requisite. Based on this finding, tourism practitioners need to boost anticipated positive emotion and lower anticipated negative emotion pertinent to international traveling as this effort minimizes the impact of the predictor on international-tourism avoidance behavior. This research contains a few limitations. First, the present study includes an issue of high correlation. Although the between-construct correlations in this study are not at the problematic level, some correlations are still high. Future research should minimize this issue by effective design of the measurement framework. Second, this research centers on the role of risk perception in eliciting emotional and behavioral responses among international travelers. Yet, according to recent destination studies in tourism, many crucial factors influence such affective and behavioral processes (e.g., satisfaction, image, destination performance, attributes, trust, value, travel experience) [1,47,48,49]. Future research should expand the proposed theoretical framework to better account for the total variance of avoidance/hesitation behavior. Such effort would enhance the comprehensiveness of the conceptual framework and its prediction power.

## Figures and Tables

**Table 1:** Measurement model and data quality assessment results (*n* = 429).

Constructs	(1)	(2)	(3)	(4)	(5)	(6)	(7)	(8)	(9)	(10)
(1) Human crowding risk	1.000	–	–	–	–	–	–	–	–	–
(2) Spatial crowding risk	0.523 ^a^ (0.274) ^b^	1.000	–	–	–	–	–	–	–	–
(3) Quality risk	0.223 (0.050)	0.363 (0.132)	1.000	–	–	–	–	–	–	–
(4) Psychological risk	0.403 (0.162)	0.412 (0.170)	0.380 (0.144)	1.000	–	–	–	–	–	–
(5) Health and safety risk	0.378 (0.143)	0.431 (0.186)	0.375 (0.141)	0.905 (0.819)	1.000	–	–	–	–	–
(6) Financial risk	0.235 (0.055)	0.375 (0.141)	0.415 (0.172)	0.456 (0.208)	0.501 (0.251)	1.000	–	–	–	–
(7) Positive anticipated emotion	−0.269 (0.072)	−0.246 (0.061)	−0.145 (0.021)	−0.564 (0.318)	−0.569 (0.324)	−0.217 (0.047)	1.000	–	–	–
(8) Negative anticipated emotion	0.159 (0.025)	0.251 (0.063)	0.209 (0.044)	0.528 (0.279)	0.573 (0.328)	0.336 (0.113)	−0.649 (0.421)	1.000	–	–
(9) Destination attachment	−0.262 (0.069)	−0.207 (0.043)	−0.080 (0.006)	−0.533 (0.284)	−0.532 (0.283)	−0.180 (0.032)	0.743 (0.552)	−0.482 (0.232)	1.000	–
(10) Avoidance/Hesitation behavior	0.363 (0.132)	0.338 (0.114)	0.238 (0.057)	0.751 (0.564)	0.780 (0.608)	0.317 (0.100)	−0.674 (0.454)	0.642 (0.412)	−0.631 (0.398)	1.000
Mean	5.664	5.117	4.622	5.677	5.601	4.944	3.385	4.759	3.045	5.509
Standard deviation	1.147	1.170	1.624	1.269	1.290	1.423	1.539	1.383	1.603	1.367
CR	0.925	0.831	0.955	0.946	0.959	0.914	0.957	0.885	0.969	0.967
AVE	0.804	0.622	0.913	0.853	0.886	0.841	0.846	0.720	0.913	0.853

Note1: ^a^ Correlations between variables are below the diagonal. Note2: ^b^ Squared correlations between variables are within parentheses. Note3: Goodness-of-fit statistics: χ^2^ = 969.977, *df* = 387, *p* < 0.001, χ^2^/d*f* = 2.506, RMSEA = 0.059, CFI = 0.963, IFI = 0.963, TLI = 0.956.

**Table 2:** Structural equation modeling results and hypothesis testing (*n* = 429).

Hypothesized Paths				Standardized Estimates	*t*-Values
H1	Risk perception	→	Positive anticipated emotion	−0.614 **	−7.430
H2	Risk perception	→	Negative anticipated emotion	0.635 **	7.327
H3	Risk perception	→	Avoidance/hesitation behavior	0.555 **	7.253
H4	Positive anticipated emotion	→	Avoidance/hesitation behavior	−0.237 **	−5.659
H5	Negative anticipated emotion	→	Avoidance/hesitation behavior	0.202 **	4.494
	Risk perception	→	Human crowding risk	0.419 **	-
	Risk perception	→	Spatial crowding risk	0.457 **	6.198
	Risk perception	→	Quality risk	0.405 **	6.145
	Risk perception	→	Psychological risk	0.961 **	8.451
	Risk perception	→	Health and safety risk	0.987 **	8.463
	Risk perception	→	Financial risk	0.531 **	6.861
**Total Variance Explained:**			**Indirect Impact on Retention:**	**Total Impact on RI:**	
R^2^ for avoidance/hesitation behavior = 0.745 R^2^ for positive anticipated emotion = 0.378 R^2^ for negative anticipated emotion = 0.403 R^2^ for human crowding risk = 0.176 R^2^ for spatial crowding risk = 0.209 R^2^ for quality risk = 0.164 R^2^ for psychological risk = 0.924 R^2^ for health and safety risk = 0.975 R^2^ for financial risk = 0.282			β_risk perception→positive & negative anticipated emotions→avoidance/hesitation behavior_ = 0.274 **	β_risk perception_ = 0.828 ** β_positive anticipated emotion_ = −0.237 ** β_negative anticipated emotion_ = 0.202 **	

Note1. Goodness-of-fit statistics for the structural model (higher-order framework): χ^2^ = 1079.063, *df* = 337, *p* < 0.001, χ^2^/*df* = 3.202, RMSEA = 0.072, CFI = 0.946, IFI = 0.946, TLI = 0.939. Note2. ** *p* < 0.01.

**Table 3:** Baseline and invariance model assessment results.

**Paths**	**High Group of Destination Attachment (*n* = 198)**		**Low Group of Destination Attachment (*n* = 231)**		**Baseline Model** **(Freely Estimated)**	**Nested Model** **(Constrained to Be Equal)**
	β	t-Values	β	t-Values		
Risk perception → Positive anticipated emotion	−0.297 **	−3.014	−0.481 **	−3.729	χ^2^ (693) = 1567.221	χ^2^ (694) = 1571.109 a
Risk perception → Negative anticipated emotion	0.416 **	3.536	0.588 **	3.883	χ^2^ (693) = 1567.221	χ^2^ (694) = 1569.933 b
Risk perception → avoidance/hesitation behavior	0.610 **	4.186	0.477 **	3.741	χ^2^ (693) = 1567.221	χ^2^ (694) = 1567.993 c
**Chi-square Difference Test:**						
^a^ Δχ^2^ (1) = 3.888, *p* < 0.05 (H6a—supported) ^b^ Δχ^2^ (1) = 2.712, *p* > 0.05 (H6b—not supported) ^c^ Δχ^2^ (1) = 0.772, *p* > 0.05 (H6c—not supported)						

Note1. Goodness-of-fit statistics for the baseline model: χ^2^ = 1567.221, *df* = 693, *p* < 0.001, χ^2^/*df* = 2.262, RMSEA = 0.054, CFI = 0.925, IFI = 0.925, TLI = 0.918; Note2. ** *p* < 0.01.

## Data Availability

The dataset used in this research are available upon request from the corresponding author. The data are not publicly available due to restrictions, i.e., privacy or ethics.
